# Antibacterial activity of *Artemisia nilagirica *leaf extracts against clinical and phytopathogenic bacteria

**DOI:** 10.1186/1472-6882-10-6

**Published:** 2010-01-29

**Authors:** Abdul R Ahameethunisa, Waheeta Hopper

**Affiliations:** 1 Department of Bioinformatics, School of Bioengineering, SRM University, Kattankulathur, Tamil Nadu, India

## Abstract

**Background:**

The six organic solvent extracts of *Artemisia nilagirica *were screened for the potential antimicrobial activity against phytopathogens and clinically important standard reference bacterial strains.

**Methods:**

The agar disk diffusion method was used to study the antibacterial activity of *A. nilagirica *extracts against 15 bacterial strains. The Minimum Inhibitory Concentration (MIC) of the plant extracts were tested using two fold agar dilution method at concentrations ranging from 32 to 512 μg/ml. The phytochemical screening of extracts was carried out for major phytochemical derivatives in *A. nilagirica*.

**Results:**

All the extracts showed inhibitory activity for gram-positive and gram-negative bacteria except for *Klebsiella pneumoniae, Enterococcus faecalis *and *Staphylococcus aureus*. The hexane extract was found to be effective against all phytopathogens with low MIC of 32 μg/ml and the methanol extract exhibited a higher inhibition activity against *Escherichia coli, Yersinia enterocolitica, Salmonella typhi*, *Enterobacter aerogenes*, *Proteus vulgaris*, *Pseudomonas aeruginosa *(32 μg/ml), *Bacillus subtilis *(64 μg/ml) and *Shigella flaxneri *(128 μg/ml). The phytochemical screening of extracts answered for the major derivative of alkaloids, amino acids, flavonoids, phenol, quinines, tannins and terpenoids.

**Conclusion:**

All the extracts showed antibacterial activity against the tested strains. Of all, methanol and hexane extracts showed high inhibition against clinical and phytopathogens, respectively. The results also indicate the presence of major phytochemical derivatives in the *A. nilagirica *extracts. Hence, the isolation and purification of therapeutic potential compounds from *A. nilagirica *could be used as an effective source against bacterial diseases in human and plants.

## Background

*Artemisia *is one of the diverse genera of *Asteraceae *family with many important medicinally valuable essential oils and secondary metabolites. Essential oils of *Artemisia *spp. have been widely used for a variety of medicinal purposes for many years. *Artemisia nilagirica *(Clarke) pamp commonly called Indian wormwood, is widely found in the hilly areas of India. *A. nilagirica *has been reported to exhibit insecticidal activities [1]. Around 59 compounds were identified from essential oil of *A. nilagirica *which showed an inhibitory activity on *Phytophthora capsici*, causing "foot rot" in pepper [2].

Various species of *Artemisia *have been characterized for their biological activities. It is considered to produce most medicinally important secondary metabolites [3,4]. Several interesting studies using *Artemisia *spp. showed a series of antimicrobial and antioxidant activities [5-9]. The qualitative determination of various secondary metabolites like flavonoids, terpenoids, saponins and polysaccharides of *Artemisia *spp. were detected by HPLC, GC-MS and NMR [10,11]. Few considerable secondary metabolites were successfully isolated and used in food industry as an alternative to synthetic antimicrobials [12,13]. Furthermore, extracts of *Artemisia *spp. were used as natural pesticide and also in the treatment of few human diseases [14-17]. The determination of potential antimicrobial activity of *Artemisia nilagirica *extracts could be more informative for the future use in controlling phytopathogens and also in clinical treatment as natural antimicrobial agents.

The organisms like *Escherichia, Enterobacter*, *Klebsiella, Proteus, Shigella *and *Staphylococcus *species are implicated to cause severe infections in human, as they are found in multiple environmental habitats [18,19]. *Erwinia *spp*.*, *Clavibacter michiganense, Pseudomonas syringae *and *Xanthomonas campestris *were reported to be severe phytopathogens, causing damage in carrot, potato, tomato, leafy greens, onion, green pepper, squash and other cucurbits. Furthermore, these phytopathogens cause disease in any plant tissue it invades [20].

In the present study, the antimicrobial potency of chloroform, diethyl ether, ethanol, hexane, methanol and petroleum ether extracts of *Artemisia nilagirica *was investigated. The antibacterial activity was determined by disk diffusion method and minimum inhibitory concentration (MIC) test. Four plant pathogens and 11 clinically important CLSI [21] reference bacterial strains from American Type Culture Collection (ATCC), Microbial Type Culture Collection (MTCC) and a local isolate from SPIC Science Foundation Patholab (SSFP) were used as test cultures. The preliminary phytochemical screening was carried out to identify the derivatives in the extracts.

## Methods

### Plant material and extraction

Fresh leaves of *Artemisia nilagirica *were collected from Nilgiri district, Tamil Nadu, India. Plant leaves were cleaned with deionized water and dried at shade for a week. Blotted leaves were grounded and filtered using four layers of gauss cloth. The plant powder was stored in air tight container and maintained at 4°C until use.

Solvent systems used for the extractions were chloroform, diethyl ether, ethanol, hexane, methanol and petroleum ether. Soxhlet and flask extraction procedures were adapted for extraction. Ten grams of the powered samples were packed in muslin cloth and used for extraction by soxhlet apparatus at a temperature below the boiling temperature of each solvent. A portion of the powdered plant samples was soaked in the conical flask containing solvent, wrapped with aluminum foil and placed in shaker for 48 hours at 120-130 rpm.

After 48 hours, the extracts were filtered using Whatman filter paper No: 1. The solvent was evaporated and the residue was dissolved in sterile dimethylsulfoxide (DMSO-9:1) in 50 mg/ml concentration. The extract was filtered using 0.22 micro filter (Type GV- Millipore) and stored at 4°C for further antimicrobial activity study.

### Test microorganisms

The 15 bacterial cultures of both gram-positive and gram-negative bacterial strains used for screening are: *Erwinia *sp. (MTCC 2760), *Xanthomonas campestris *(MTCC 2286), *Pseudomonas syringae *(ATCC 7386), *Clavibacter michiganense *(ATCC 27822), *Escherichia coli (*ATCC 25922), *Yersinia enterocolitica *(MTCC 840), *Klebsiella pneumoniae *(ATCC 15380), *Salmonella typhi *(SSFP 4S), *Enterobacter aerogenes (MTCC 111), Proteus vulgaris (MTCC 1771), Pseudomonas aeruginosa *(ATCC 27853), *Shigella flaxneri (MTCC *1457), *Bacillus subtilis *(MTCC 441), *Enterococcus faecalis *(ATCC 29212) and *Staphylococcus aureus (MTCC 29212)*. The stock cultures were maintained in nutrient agar (NA) slant at 4°C and sub-cultured monthly. Working cultures were prepared by inoculating a loopful of each test microorganism in 3 ml of nutrient broth (NB) from NA slants. Broths were incubated at 37°C for 12 hours. The suspension was diluted with sterile distilled water to obtain approximately 10^6 ^CFU/ml.

### Screening for antibacterial activity

Disk diffusion: 5 mm of sterile disks were incorporated in 100 μl of plant extracts (5 mg/disk). The disk was completely saturated with the extract and allowed to dry. Mueller Hinton (MH) agar plates were swabbed with test bacteria and six extract disks with one of the standard positive control disks (ampicillin, streptomycin or gentamycin) was placed on the MH agar plate. DMSO was taken as the negative control. Plates were incubated overnight at 37°C.

### Agar dilution susceptibility test

The agar dilution susceptibility test was performed based on modified method of NCCLS and CLSI [22,23] to determine the MIC. Extracts dissolved in sterilized DMSO (5120 μg/ml concentration) were taken as standard stock. A series of two fold dilutions of each extract in the final concentration of 512, 256, 128, 64 and 32 μg/ml were prepared in MH agar. After solidification, the plates were spotted with 2 μl of overnight grown bacterial cultures approximately containing 1 × 10^4 ^CFU/ml. The test was carried out in triplicates. The plates were incubated overnight at 37°C. After 18 - 24 hours, the MIC was determined and the percentage of growth inhibition was calculated by,

T: Test: SC: Solvent control; PC: positive control

### Phytochemical screening

To identify the phytochemical derivatives in the extracts, standard phytochemical screening was performed [24,25]. Alkaloids test was performed by Dragendorff's and Meyer's tests, amino acids by ninhydrin, carbohydrates by Barfoed's and Fehling tests, flavonoids by FeCl_3_, Pew's and Shinoda's tests, glycosides by Killer-Killanis test, saponin by frothing test, tannins by FeCl_3 _and lead acetate & terpenoids by Salkowski test. The test for hydrolysable tannins, phlobatannins, phenol, quinones and volatile oils were also carried out as in literature [26-28].

### Statistical analysis

Analysis of variance (ANOVA) was performed using GeneSpring GX - 7.3 microarray software to determine the significance of MIC values between the extracts against bacterial culture. The probability value (p) ≤ 0.05 was considered as significance in this study.

## Results & Discussion

The antibacterial activity of *A. nilagirica *leaf extracts were examined against 11 clinical and four phytopathogens causing illness in human and damage in major crops [20], respectively. The extractions were carried out using chloroform, diethyl ether, ethanol, hexane, methanol and petroleum ether solvents. The ethanol and methanol extracts gave the high yield of 2.3% (% concentration w/v) and hexane gave 1.6% w/v. While, other extracts provide much low yield of 0.6% w/v in soxhlet and flask extraction procedures. The antibacterial activity of the organic solvent extracts showed varying magnitudes of inhibition patterns with standard positive control depending on the susceptibility of the tested microorganism. Out of 15 bacterial strains tested, 12 showed inhibition activity to one or more extracts. The mean inhibitory zone of six solvent extracts against 15 bacterial species is summarized in Table 1.

**Table 1:** Antibacterial activity screening of *A. nilagirica *by disk diffusion

	Zone of inhibition (mm diameter)							
**Bacterial culture**	**Positive control**	**Solvent control**	**Ethanol**	**Methanol**	**Hexane**	**Petroleum Ether**	**Chloroform**	**Diethyl ether**

*Erwinia sp*.	15	-	10 ± 0.0	12 ± 0.5	13 ± 1.0	10 ± 0.0	12 ± 0.0	10 ± 0.5

*X. campestris*	16	-	11 ± 0.5	12 ± 0.0	14 ± 0.0	11 ± 0.5	12 ± 0.1	11 ± 0.5

*P. syringae*	14	-	11.8 ± 0.5	11 ± 0.0	12 ± 0.0	10 ± 0.0	10 ± 0.0	12 ± 0.5

*C. michiganense*	15	-	12 ± 1.0	12 ± 0.0	13 ± 0.0	8 ± 0.0	11 ± 0.0	12.4 ± 0.5

*E. coli*	13	-	14 ± 1.0	12 ± 0.5	8 ± 0.0	8 ± 0.0	11 ± 0.5	11 ± 0.0

*Y. enterocolitica*	14	-	11 ± 1.0	12 ± 0.0	11 ± 0.0	9 ± 0.5	13 ± 0.0	14 ± 0.0

*B. subtilis*	13	-	8 ± 0.0	8 ± 0.0	-	8 ± 0.0	10 ± 0.0	10 ± 0.0

*E. faccalis*	14	-	-	-	-	-	7 ± 0.0	8 ± 0.0

*K. pneumonia*	12	-	-	8 ± 0.0	-	-	-	-

*S. typhi*	10	-	8 ± 0.0	10 ± 0.0	13 ± 1.0	12 ± 1.0	8 ± 0.0	8 ± 0.0

*S. aureus*	12	-	-	-	-	-	7 ± 0.0	-

*E. acrogens*	14	-	8 ± 0.0	11 ± 1.0	8 ± 0.0	8 ± 0.0	10 ± 0.0	8 ± 0.5

*P. valgaris*	14	-	8 ± 0.0	12 ± 1.0	14 ± 0.0	14 ± 0.5	10 ± 0.0	8 ± 0.0

*P. aeruginosa*	13	-	10 ± 0.0	12 ± 0.0	13 ± 0.0	12 ± 0.0	10 ± 0.0	8 ± 0.0

*S. flexneri*	14	-	8 ± 0.0	10 ± 1.0	8 ± 0.0	8 ± 0.0	10 ± 0.0	8 ± 0.0

± - mean standard deviation of triplicates, Concentration of extract- 5 mg/disk, (-) - No zone of inhibition observed, Positive controls - ampicillin or streptomycin or gentamycin (10 μg/ml), Solvent control - 10% DMSO.

The analysis of methanol and chloroform extracts against phytopathogens showed a significant level of inhibition against *Erwinia *sp. and *X. campestris*. On the other hand, ethanol and diethyl ether extracts showed high activity against *C. michiganense *and *P. syringae *(p ≤ 0.05). Also, the petroleum ether extracts showed 11-12 mm zone of inhibition to *C. michiganense*. Interestingly, hexane extract of *A. nilagirica *exhibits maximum inhibitory activity against all the phytopathogens in comparison to other extracts [Table 1]. Further, hexane extracts showed the significant inhibitory effect against *Clavibacter michiganense *(13 mm), *Erwinia *sp (13 mm), *Pseudomonas syringae *(12 mm) and *Xanthomonas campestris *(14 mm). It is understandable that hexane extract is more potent showing a higher degree of antimicrobial activity to phytopathogens in comparison to other extracts. Also, the results of hexane extract against *X. campestris *was appeared to be two-fold better than the previous study of *Phyllanthus emblica, Acacia nilotica, Sapindus mukorossi *and *Terminalia chebula *which shows 6.00 mm zone of inhibition at 50 gm/l concentration reported as the most effective for *X. campestris *[29]. In addition, moderate effects were seen in chloroform, diethyl ether, ethanol and methanol extracts against all tested phytopathogens except petroleum ether which showed comparatively minimum area of inhibition. This possibly means that the compound responsible for the antibacterial activity was least in concentration.

Similar analysis of *A. nilagirica *leaf extracts were carried out on clinical bacterial pathogens. The hexane, methanol and petroleum ether extracts exhibited significant high inhibitory zones against *P. aeruginosa, P. vulgaris *and *S. typhi*. The chloroform and diethyl ether extracts showed maximum area zone of inhibition (10 mm) for *B. subtilis*, compared to other extracts (8 mm). The ethanol extract exhibited 14 mm zone for *E. coli*, which is the maximum with respect to the positive standard streptomycin. Subsequently, antibacterial activity of methanol (12 mm), chloroform (13 mm) and diethyl ether (14 mm) extracts were found as effective for *Y. enterocolitica*. Moderate activities were observed against *S. flexneri *and *E. aerogenes *for chloroform and methanol extracts. Among the 11 clinical bacterial strains, *Escherichia coli, Yersinia enterocolitica, Bacillus subtilis, Salmonella typhi, Enterobacter aerogenes, Proteus vulgaris, Pseudomonas aeruginosa *and *Shigella flexneri *were the most susceptible bacteria to all solvent extracts. Nearly, 40% growth inhibitions were observed in concentration of 5 mg/ml. Surprisingly, no activity were observed against *S. aureus, E. faecalis *and *K. pneumoniae *suggesting their resistance to *A. nilagirica *extracts.

The MIC tests of *A. nilagirica *organic solvent extracts against 15 bacterial species were carried out using the micro dilution technique. The MIC values of 6 extracts ranged from 32 to 512 μg/ml [Table 2]. While considering phytopathogens, chloroform extract showed maximum activity with MIC 32 μg/ml for *Erwinia *sp. and *X. campestris*. Diethyl ether extracts showed 32 μg/ml MIC for *P. syringae *and *C.michiganense*. In general, all extracts showed less than 128 μg/ml MIC for the tested phytopathogens. Interestingly, hexane extract had higher activity in all the phytopathogens even at a low concentration of 32 μg/ml. The MIC analysis of clinical pathogens showed that the methanol extract was highly active in comparison to other extracts, which inhibited the series of study organisms at a low concentration (32 μg/ml) except *B. subtilis *(64 μg/ml) and *Shigella flexneri *(128 μg/ml) [Table 2]. In supportive to the susceptible test, *A. nilagirica *extracts had very low or no activity, even in the highest concentration (512 μg/ml) MIC against *S. aureus*, *E. faecalis *and *K. pneumoniae*. Hence, we conclude that these organisms are resistant to *A. nilagirica *extracts. According to the results from zone of inhibition and MIC studies [Table 1 &2], hexane and methanol extracts are considerably good inhibitors for the phytopathogens and clinical pathogenic bacteria, respectively.

**Table 2:** Minimum inhibitory concentration (MIC) of various solvent extracts of *A. nilagirica *(% v/v) against micro-organisms

Test organism	Plant extracts						
		**Chloroform**	**Diethyl ether**	**Ethanol**	**Hexane**	**Methanol**	**Petroleum ether**

		**Concentration **(μg/ml) showing Minimum inhibitor concentration					

*Erwinia sp*.	Phytopathogens	32	64	64	32	64	64
		
*Xanthomonas campestris*		32	64	32	32	32	128
		
*Pseudomonas syringae*		64	32	128	32	64	64
		
*Clavibacter michiganense*		128	32	32	32	32	32

*Escherichia coli*	Clinical pathogens	256	256	32	256	32	128
		
*Yersinia enterocolitica*		64	256	128	128	32	512
		
*Basillus subtilis*		32	32	32	32	64	32
		
*Enterococcus faccalis*		*	*	*	*	512	*
		
*Klebsiella pnumonia*		*	*	*	*	*	*
		
*Salmonella typhi*		32	64	128	64	32	32
		
*Staphylococcus aureus*		*	*	*	512	*	*
		
*Entrobacter acrogens*		512	128	32	128	32	32
		
*Proteus valgaris*		32	256	128	32	32	64
		
*Pseudomonas aeruginosa*		512	128	128	128	32	512
		
*Shigella flexneri*		512	256	256	256	128	128

* No inhibition observed upto: 512 μg/ml.

Phytochemical test were carried out on the *A. nilagirica *extracts to determine the natural bioactive compound. By studying the presence of phytochemical in *A. nilagirica*, the medicinal value of the plant can be explained scientifically. The phytochemical screening of extracts showed the presence of major derivatives and their results were summarized [Table 3]. The analysis showed the occurrence of alkaloids, flavonoids, phenol, quinines and terpenoids in all extracts. Tannins were present in ethanol, methanol and diethyl ether. Volatile oils were present in methanol, hexane and petroleum ether. Phlobatannins metabolites were found to be present in hexane and petroleum ether and absent in other extracts. Also, saponins and amino acid were present in ethanol and methanol extracts with carbohydrates particularly present in methanol extract. Surprisingly, glycosides and hydrolysable tannins were absent in all the extracts.

**Table 3:** Phytochemical screening of *A. nilagirica *extracts

**S. no**.	Test	Chloroform	Diethyl ether	Ethanol	Hexane	Methanol	Petroleum ether
1	Alkaloids	++	++	++	++	++	+
2	Amino acids	--	--	+	--	++	--
3	Carbohydrates	--	--	--	--	+	--
4	Flavonoids	++	+	++	++	++	+
5	Glycosides	--	--	--	--	--	--
6	Hydrolysable tannins	--	--	--	--	--	--
7	Phenol	++	+	++	+	++	+
8	Phlobatannins	--	--	--	+	--	+
9	Quinines	+	+	+	+	+	+
10	Saponin	--	--	+	--	+	--
11	Tannins	--	+	++	--	++	--
12	Terpenoids	++	++	++	++	++	++
13	Volatile oils	--	--	--	+	+	--

(++) abundant, (+) present, (--) absent

In susceptibility test, the hexane extracts showed the considerable levels of inhibition against phytopathogens. The phytopathogens test of petroleum ether extract showed low inhibition range (8 to 10 mm) in comparison to other extracts (10 to 14 mm). In conjugation with phytochemical screening of all the extracts with petroleum ether, showed the variations in abundance in alkaloids derivates. Hence, it is suggested that reduction of alkaloid abundance in petroleum ether may be the cause of decreased activity in phytopathogens. Supportive to our finding, previous studies indicate the effective role of alkaloid against phytopathogens [30-32]. The MIC analyses of clinical pathogens showed an activity against Gram-positive and Gram-negative bacteria may be indicative of the presence of the broad spectrum antibiotic compounds. The methanol extracts showed high inhibition at the minimal concentration for most of the clinical pathogens in comparison to other extracts. The MIC value of methanol extract ranges from 32 to 64 μg/ml for *Escherichia coli, Bacillus subtilis, Yersinia enterocolitica, Salmonella typhi, Enterobacter aerogenes, Proteus vulgaris *and *Pseudomonas aeruginosa*. Also, the phytochemical screening of menthol extract showed the presence of most of the derivatives like flavonoids, terpenoids, phenol, amino acids, alkaloids and tannins. Furthermore, alkaloids [33,34], amino acids [35], flavonoids [36-38], phenols [39], tannins [40-42], terpenoids [43] of various plants extracts proven to be effective antimicrobials [44]. Our results are also in agreement with these studies suggesting the efficacy of methanol extract of *A. nilagirica *against clinical pathogens.

## Conclusion

Extracts of *A. nilagirica *showed the broad spectrum of antibacterial activity on the tested microorganisms. Hexane extract exhibited high inhibitory potency against phytopathogens and methanol extract showed maximum inhibition against clinical pathogens except *S. aureus*, *E. faecalis and K. pneumoniae*. The phytochemical analysis showed the presence of effective biological compounds like alkaloids, amino acids, flavonoids, phenols, tannins and terpenoids. These derivatives could be potential alternatives to the traditional chemical control of clinical pathogen and phytopathogenic bacteria. Furthermore, the development of natural antimicrobials will help to decrease the negative effects of synthetic drugs. Fractionation and characterization of these active compounds will be the future work to investigate.

## Competing interests

The authors declare that they have no competing interests.

## Authors' contributions

AA has carried out the experimental part such as extraction, inoculums preparation and antimicrobial evaluation. WH supervised the work, evaluated the results and corrected the manuscript for publication. Authors read and approved the final manuscript.

## Pre-publication history

The pre-publication history for this paper can be accessed here:

http://www.biomedcentral.com/1472-6882/10/6/prepub
